# Physician Perspectives on Web-Based Real-World Statistics for Better-Informed Drug Selection in Epilepsy: Mixed Methods Study

**DOI:** 10.2196/82958

**Published:** 2026-02-11

**Authors:** David Larsson, André Idegård, Samuel Håkansson, Johan Zelano

**Affiliations:** 1 Department of Neurocare, Member of ERN EpiCARE, Sahlgrenska University Hospital, Gothenburg, Sweden; 2 Wallenberg Center for Molecular and Translational Medicine, University of Gothenburg, Gothenburg, Sweden; 3 Department of Clinical Neuroscience, Institute of Neuroscience and Physiology, University of Gothenburg, Blå stråket 7, Gothenburg, 41345, Sweden, 46 313420000; 4 Department of Health Sciences and Technology, ETH Zürich, Zürich, Switzerland

**Keywords:** seizure, quality improvement, health care innovation, antiepileptic drug, retention rate, antiseizure medication discontinuation, ASM discontinuation

## Abstract

**Background:**

Lately, big data studies have shown promise in using patient characteristics to rank the likelihood of retention of antiseizure medications (ASMs), a measure indicating tolerability as well as effect. How such results can be integrated into clinical practice has yet to be studied. We developed EPstat, a noncommercial tool that provides physicians with real-world treatment retention data from 33,998 patients with epilepsy.

**Objective:**

This study investigated the user experience of EPstat after its pilot launch.

**Methods:**

EPstat was developed in an iterative process with first a prototype and then a final version accessible on the health care region intranet. EPstat was launched in 2022 through emails and information meetings at neurology departments. After 1 year, an online questionnaire was distributed to physicians in our health service region’s neurology clinics (5 hospitals). Descriptive statistics and thematic analysis were used to summarize responses. To supplement the survey, 3 semistructured workshops or group interviews with neurologists and residents were used to gather further feedback.

**Results:**

Of the 27 survey respondents, 19 (70%) were aware of EPstat and 10 (37%) had used it. Users rated EPstat highly for ease of use (median 5, IQR 4‐5) and applicability in clinical practice (median 4, IQR 4‐4). Two of the 10 respondents who had used it indicated that the platform had influenced their choice of ASM. Workshop participants advocated for expanding the platform to include retention data on newer ASMs and general information relevant to epilepsy management.

**Conclusions:**

The notion of using big data to improve ASM selection was well received. However, there were barriers to the initial use, and users requested a more comprehensive resource that also incorporated other information related to epilepsy. EPstat is now being updated with more recent ASM statistics, including information on newer ASMs. Mobile access, more information for physicians, and mentioning the tool in regional guidelines are some possible measures to increase use. Linking multinational statistics could also increase the precision of the presented data and, thus, increase usefulness. Study of EPstat will continue and should include thematic analysis of representative and rigorously sampled workshop participants. Such studies are also likely to provide information on how physicians and health services receive web-based tools, which are likely to soon be driven by artificial intelligence. In similar projects, we recommend greater participatory involvement of both health care providers and patients already at the design stage.

## Introduction

Antiseizure medication (ASM) selection is a fundamental aspect of epilepsy care. A key challenge is the availability of over 25 ASMs, each with a different mechanism of action and side effect profile, and the related difficulty of selecting the most appropriate drug for each individual patient. Current decision-making mainly relies on clinical trials and guidelines that may not fully capture the diversity of real-world clinical scenarios. For instance, the most commonly prescribed first ASM in Sweden is levetiracetam [1,2], although there is randomized controlled trial evidence suggesting that lamotrigine is equally efficacious with lower risk of side effects [3]. This discrepancy highlights a gap between available evidence and everyday clinical practice.

Ideally, when deciding which ASM to prescribe, the type of epilepsy indicates a range of suitable options, with the final choice being based on patient-specific factors such as presumed side effect sensitivities, comorbidities, drug interactions, and patient preference. ASMs are typically approved after clinical trials have demonstrated an effect on seizures and assessed short-term safety, but these trials often lack the necessary details to guide personalized treatment. Indeed, observational data suggest that the tolerability of certain ASMs may vary across different age groups and epilepsy etiologies [4,5].

Recently, big data sources such as administrative registers or claims data have been used to track ASM use in thousands of patients [5,6], which allows for stratification by patient characteristics. Retention rate, an often-used measure in epilepsy trials integrating effect and tolerability, can be captured in administrative data [7]. For instance, we have used prescription data to track ASM use in Swedish patients and shown that lamotrigine and levetiracetam have superior retention rates to that of carbamazepine in patients developing epilepsy after stroke [8], which agrees with small randomized trials in the same patient group [9,10]. In subsequent studies, we have demonstrated that age, sex, and comorbidities affect which ASM has the highest likelihood of retention, indicating a need for more personalized ASM selection [5]. Lamotrigine has the highest retention rate in focal epilepsy, providing real-world support for the results from large randomized trials [11]. Interestingly, retention rates in Swedish prescription data also agree closely with expert advice on which ASM suits which patient [12].

Big data on epilepsy hold clear potential for personalized medicine and tailored management; by tracking ASM use and assessing drug retention in specific patient groups, it becomes possible to determine which ASM has the highest probability of success for individuals across different age groups and those with various comorbidities and concurrent medications. In the future, artificial intelligence (AI)–based suggestions of ASM selection are not unlikely [13], making it important to study how big data tools are received in everyday health care.

**Figure 1.**
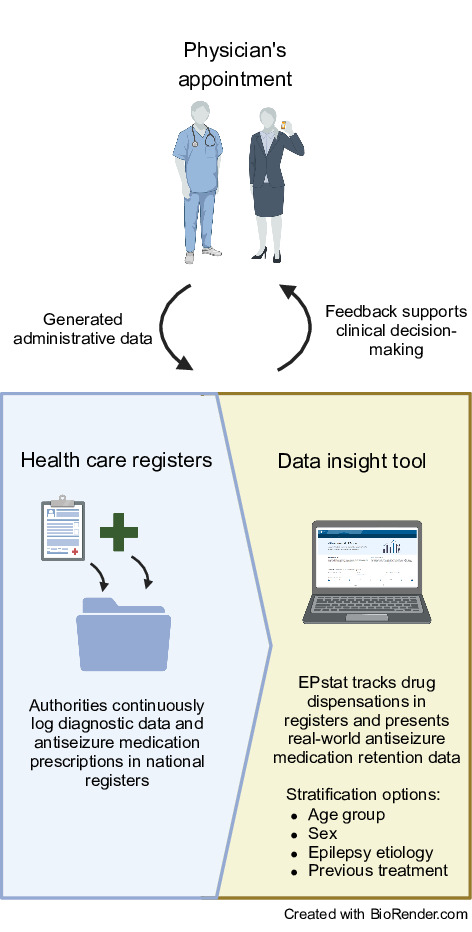
Schematic of EPstat concept. Data from routine health care registers are processed to generate statistics on retention rates. This is accessible through a website, thereby providing real-world treatment retention rates, supporting clinicians in the selection of antiseizure medications.

In a regional innovation project aimed at improving ASM selection by looping data from routine health care into the decision-making process, we developed a web-based resource called EPstat designed to assist in selecting first and second ASMs (Figure 1). To evaluate the perceived value of the tool, we conducted a cross-sectional survey and held semistructured workshops with neurologists and neurology residents in our region.

## Methods

We used a mixed methods approach comprising a quantitative cross-sectional survey evaluating EPstat’s user experience and clinical applicability and a series of qualitative workshops to gather suggestions for potential improvements to the platform. The manuscript follows the Standards for Quality Improvement Reporting Excellence reporting guidelines [14].

### Ethical Considerations

The EPstat project was approved by the Ethical Review Authority (approval 2022-214). No personal data were processed; thus, according to Swedish law, this study did not require consent. There was no compensation given to participants.

### About EPstat

The EPstat platform is web-based and noncommercial and was conceptualized by clinicians in close collaboration with the IT department of Region Västra Götaland (health care provider for 1.7 million inhabitants). The design of the website presenting the underlying research results was iterated through interviews with clinicians outside the research group (prototype), and once a suitable concept was obtained, the website was built by the IT department in collaboration with DL and JZ (both neurologists). It was launched in 2022 through information meetings at 5 neurology clinics (1 tertiary center and 4 regional hospitals). After introductory meetings with clinicians, they were encouraged to try out the tool themselves during their routine clinical duties and were informed that they could contact the responsible parties if they had any questions. EPstat is currently only accessible on computers within the health care provider’s network because the project is still in its concept development stage.

The information on the platform is based on nationwide health care administrative data from encounters with specialized care, including all neurology clinics and all dispensations of prescription drugs at pharmacies. It includes ASM retention rates based on data from 33,998 patients with epilepsy onset after the age of 25 years (presumed focal epilepsy) from 2007 to 2019. The cohort characteristics, the data sources, and the method for tracking ASM treatment using prescription data have been described previously [5].

EPstat’s interface is minimalistic, presenting information on ASM retention rates (including 95% CIs), which refers to the proportion of patients continuing to use the drug at a specific point in time. High treatment retention is preferable and suggests that the regimen is efficacious and tolerable for many patients receiving it. The user has the choice to use the entire cohort or stratify the information according to adjustable age groups; sex; common causes of epilepsy; or, if relevant, one previous treatment regimen. Only ASMs with an indication for focal epilepsy according to recommendations from the Swedish Medical Products Agency [15] are included; in certain situations in which a specific ASM is clearly contraindicated, such as valproic acid for women of childbearing potential, that ASM is excluded.

### Survey

In 2023, approximately 1 year after EPstat’s pilot launch, an online survey was emailed to physicians at the 5 neurology clinics to evaluate their awareness of and experiences with the platform. Approximately 100 neurologists and residents are employed at the sites, but some are unavailable at any given time due to various types of leave, such as parental, sick, or research leave. The head of each clinic forwarded the survey invitation to the employees. The survey was designed to be brief and anonymous, with main outcome measures comprising rating scales from 1 to 5 for ease of use, clinical applicability, the likelihood of recommending the platform to a colleague, and the desire for EPstat to continue to be available and maintained. We asked additional questions about whether EPstat had influenced the respondents’ choice of ASM (yes or no) or whether they felt that any functions were missing (open-ended). Key questions had mandatory fields to ensure completeness. We summarized the responses using descriptive statistics and conducted thematic analyses of the open-ended responses to identify common themes and suggestions for improvement.

### Workshops

Three workshops were conducted with author DL as the moderator. The main goal was to gather ideas for improvement of the platform. Participants comprised physicians (intended users) at the participating neurology clinics, up to 6 in each workshop. To ensure diverse perspectives, we recruited both experienced neurologists and residents early in their training. The feedback was documented and then qualitatively analyzed by DL and JZ to identify common themes. The analytic approach was qualitative based on content coding; each comment (individual or group derived, as described in the next paragraph) was labeled using descriptive codes, which were then grouped by themes.

The interviews were semistructured, starting with a brief project description, including a presentation of the survey findings, followed by a brainwriting session (brainstorming with affinity mapping) in which participants first reflected separately and then took turns sharing individual comments on paper notes, which were subsequently grouped to identify common themes, which were then developed further in the group. The participants also had the opportunity to share their opinions and thoughts about EPstat openly.

## Results

### Survey

**Figure 2.**
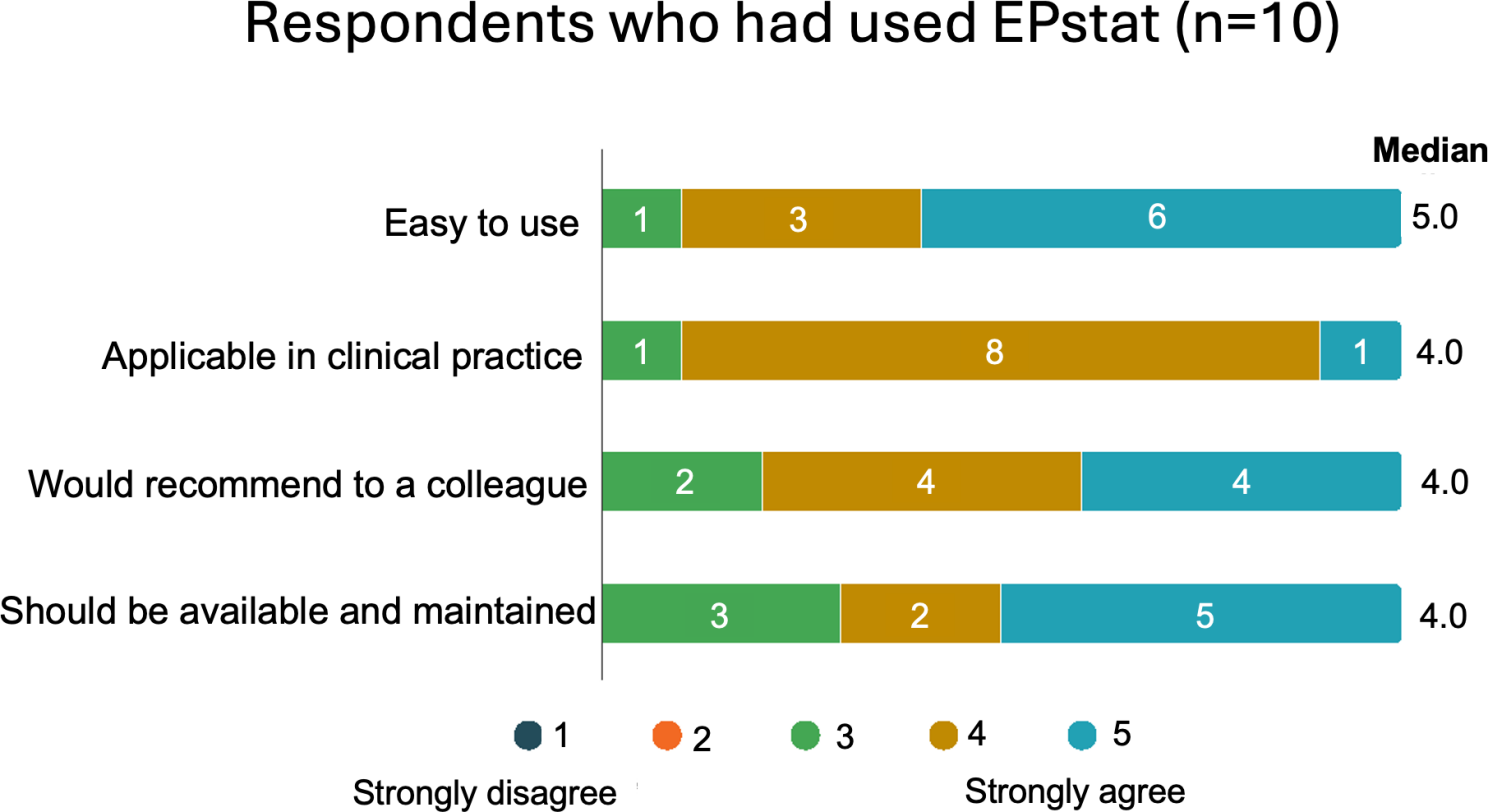
(A) Survey responses from 10 physicians who used EPstat, rating their agreement with statements about the platform’s ease of use and usefulness on a scale from 1 to 5. (B) Distribution of the survey responses from the physicians who indicated their primary reason for not using EPstat or for using it infrequently (n=19).

Of the 27 physicians who responded to the survey, 23 (85%) were neurologists, and 7 (26%) described themselves as subspecialized in epilepsy. The number of physicians who received the survey invitation is estimated to be less than 100 based on the number of physicians in the participating departments, resulting in a response rate of ≥27%. A total of 70% (19/27) were aware of the EPstat resource, but nearly half (9/19, 47%) had not used it. Among the 10 who had used the platform, it was rated highly on a scale from 1 to 5 for ease of use (median 5, IQR 4‐5) and applicability in clinical practice (median 4, IQR 4‐4). The likelihood of recommending the platform to a colleague (median 4, IQR 4‐5) and support for its continued availability (median 4.5, IQR 3‐5) was also positively rated on the same scale (Figure 2). In total, 20% (2/10) of the respondents who had used it indicated that the platform had influenced their choice of ASM. Among those who had heard of the resource but had not used it, forgetfulness was cited as the primary reason by 58% (11/19), that the results did not differ from routine clinical practice by 21% (4/19), that Epstat was used as much as needed by 10.5% (2/10).

The questionnaire also included 1 free-text question about anything they felt was missing in EPstat and another for additional comments or feedback. However, there were only 2 responses to these questions, both specifying why the respondent had not used the platform. The first respondent mentioned forgetfulness and time constraints, whereas the second noted that EPstat was probably a helpful tool but that they had not encountered the right situations to use it.

### Workshops

We conducted 3 workshops with a total of 10 participants representing 4 different neurology clinics (the tertiary center and 3 regional hospitals). Table 1 provides a summary of the feedback and identified themes. Participants generally found the platform easy to use and appreciated its appearance. However, several reported that they felt that the information provided was too basic, especially for experienced specialists. Challenges typically only arise after selecting the third or subsequent ASMs or when typical first-line drugs are unsuitable. They also observed that lamotrigine was usually the drug with the highest retention regardless of age group and other characteristics, which limited the need to visit the platform regularly. It was suggested that the platform might be more beneficial for junior physicians.

**Table 1.** Summary of feedback and identified themes from the workshops. The themes are listed in order of perceived prominence in the discussions.

Theme	Included codes	Summary
Expand data scope	Combination therapy, comorbidities, compliance, detailed patient stratification, discontinuation reasons, efficacy statistics, generalized epilepsy, less common ASMs^a^, longer retention rates, more recent data, and third ASM data	Include retention data on third ASMs and combination therapies; update the tool with recent information; provide data on long-term retention; and incorporate data on epilepsy type, various comorbidities, and characteristics that may influence drug suitability to allow for detailed patient stratification. Participants also requested information on reasons for drug discontinuation, including inefficacy, side effects, and reduced compliance.
Expand drug information	Drug information, drug interactions, pregnancy risk, seizure recurrence risk, serum concentration, side effects, steady state, and treatment guide	Include information regarding the most clinically relevant side effects and drug-drug interactions, as well as target ranges (trough levels) and timing for blood concentration tests. Participants were also interested in pregnancy-related drug information and the risk of recurrent seizures and whether it varied between different ASMs. Others advocated for a more comprehensive treatment guide with all relevant drug information compiled.
Accessibility and usability	Accessibility, data presentation, direct link in EHR^b^, exclude drugs, mobile app, retention graphs, and visual design	Ensure that the tool can be found via search engines or integrated directly into the electronic medical record system. Several participants suggested developing a mobile app version. Data could be presented in graphs, and the visual design could be improved with a more interesting color scheme, possibly allowing users to pick their preferred scheme. Additionally, the ability to exclude selected drugs would be a valuable feature.
General epilepsy educational resources	Basic epilepsy information, diagnostic guide, epilepsy surgery, guidelines, and link compilation	Include basic epilepsy information tailored to junior physicians; a diagnostic guide for identifying and classifying seizure types; and a collection of educational links to reputable sources, including current guidelines.
Practical tools for clinical use	Patient information, patient logging, prediction tool, and titration plans	Include printable patient information and standardized titration plans that can be copied and pasted. There were also suggestions for an additional feature in which patients could log seizures and side effects.
Limitations	Junior physicians, lack of resolution, misinterpretation risk, and too basic	Several participants considered the information provided too basic for their needs; there were suggestions that it might be more useful for junior physicians. Some thought that the recommendations lacked resolution, causing them to rely on other individual factors when making decisions. Additionally, overly stratifying the information could generate strange results that might be misinterpreted.
Promotion and stakeholder involvement	Involve epilepsy process team, local guidelines, promotion, and promotion to junior physicians	Suggestions included involving the regional epilepsy process team, adding information about EPstat to local guidelines for new-onset epilepsy consultations, promoting the tool during continuing education, and targeting advertisements to junior physicians.

a ASM: antiseizure medication.

b EHR: electronic health record.

Notably, requests for information on combination therapy retention and third ASM data (under the “Expand data scope” theme), information on drug interactions and side effects (under the “Expand drug information” theme), and a link compilation (under the “General epilepsy educational resources” theme) were consistently suggested during all 3 workshops. Other common suggestions discussed during at least 2 workshops included incorporating general and drug-specific patient information for printing and copying, compiling general epilepsy information, increasing accessibility by making the platform searchable through Google or other search engines, or developing an app.

## Discussion

We used a multicenter mixed methods approach to investigate how physicians experienced EPstat. Few had used it, but those who had generally perceived the platform as clinically relevant and backed its continued availability, indicating that the concept of a web-based resource to support ASM selection was well received. Notably, 2 of 10 users stated that the platform had impacted their ASM choice. However, despite the high ratings in the survey, several workshop participants thought that the information provided was too basic for a specialist’s needs and requested additional information on subsequent ASMs and combination therapy and the possibility of a more detailed stratification. They also asked for further drug information; general educational resources; and other practical resources, including patient information and standardized treatment titration schedules. Participants considered accessibility to the platform to be low and suggested that the site should be searchable online or accessible via a mobile app.

The suggestions of a gap between the platform’s current content and the needs of experienced clinicians are interesting. While several participants felt that the information provided did not differ from clinical routine, there is recent observational evidence suggesting that lamotrigine, the drug with the highest real-world retention in most subgroups, is only prescribed as the first choice in approximately one-third of all cases in our region. Therefore, it is possible that information perceived as basic could still improve management. Moreover, while there were requests for more advanced data, some participants also suggested adding information on fundamental concepts, illustrating the diverse needs among users. It is interesting that only 7% (2/27) of the respondents indicated that EPstat had an influence on their clinical decisions. This could represent early-stage barriers but also that EPstat provides information supporting routine practice. Most early users are probably epilepsy-interested physicians who know treatment guidelines, and for them, EPstat offers limited new information. As we only introduced data until 2019, there are also intrinsic limitations—we could only include older ASMs that have been on the market for a long time. As register data grow, EPstat will be able to provide information on newer ASMs. For instance, we have recently published a new retention rate study on data until 2023 [16], and these data will be incorporated into new EPstat versions. In this new version, there are enough data points to offer statistics on retention of new ASMs such as lacosamide and brivaracetam, so we hope that EPstat will be perceived as more informative for clinical decisions in the future. We also hope that EPstat will be increasingly used by junior physicians and physicians not experienced in epilepsy (the tool is now introduced in local guidelines as a resource). The survey responses indicated that forgetting about the existence of EPstat was a major reason for nonuse, so better marketing and prompting is also important. The restricted access (only within the health care provider network) could be another factor, but the effect should be minor as all outpatient rooms have permanent in-network computers that are used during patient visits for electronic health records and prescriptions.

In hindsight, it would probably have been advantageous to conduct even more participatory research in the design of EPstat. Although we did reach out to clinicians and conducted interviews during the development, we could have added other features that could have served as attractants (patient information leaflets on risk mitigation for printing, national guidelines, or similar resources). With regard to actual participatory research—involving citizens and patients—the EPstat development could also have been designed in a better manner. Culture may well be an important factor in this case; we did identify patients as stakeholders at the start of the project and had meetings with the local patient organization during development, and the reception of the tool was generally positive, the framing being that it is good if all patients in our large health region receive their selected epilepsy medication on a similar basis. EPstat was perceived as an effort to enhance the quality of care outside tertiary centers. However, Swedish health care is very strict on equality between different diseases and patient groups, and there is no strong tradition of involving stakeholders in a manner similar to other countries, where patient organizations or charities can be more directly involved. In later stages of the project, we encouraged physicians to use EPstat in patient discussions—when the patient and physician decide on which drug to choose (generally a shared decision). Thus, later in the project, patients became target users, and it would have been better to involve them as participants earlier in the project. In participatory framework terminology [17], their level of involvement in this study was low (informing). For future versions or similar endeavors, a higher level of participation (partnership) could be achieved through earlier involvement of patients and their representatives and by allocating more resources to that involvement. A higher degree of participation could probably help in disseminating EPstat use; patient questions could, for instance, spur physician engagement with the tool.

EPstat is the first clinical tool to use real-world data as feedback to support physicians in selecting ASMs. A similar tool, EpiPick, uses an algorithm that uses input from expert consensus to provide tailored ASM recommendations. The recommendations of both tools often align [12], and both contribute valuable information for clinicians. However, as the data provided by EPstat reflect actual patient outcomes, we believe that they provide complementary insights compared to expert opinions, which may be more theoretical. EPstat also offers the potential advantage of feedback from continuous health care updates from the country where the physicians practice.

When interpreting the feedback from the workshops, it becomes clear that participants want EPstat to evolve into an epilepsy go-to hub that incorporates both real-world treatment retention data and a wide range of other information. They suggested several fitting additions, such as general drug information on potential side effects, drug-drug interactions, target ranges and timing of blood concentration tests, and pregnancy-related considerations, as well as general epilepsy educational resources and patient information. However, several suggested improvements under the “Expand data scope” theme, which was prominent in the discussions, cannot be addressed as easily. Prescription data have inherent limitations, making distinguishing between monotherapy and polytherapy in the context of multiple dispensed ASMs challenging. While it may be possible to include retention data on the third and subsequent ASMs, validation against medical records would likely be necessary to confirm accuracy. More detailed patient stratification may also be possible, but administrative data typically lack disease-specific details, for example, on epilepsy severity, and some comorbidities, such as some psychiatric disorders and vascular risk factors, have low sensitivity in the national registers due to primarily being managed in primary care.

This study’s strengths lie in the active involvement of clinicians, ensuring that platform development was user-centered, and the multicenter mixed methods approach, gathering both quantitative and qualitative insights into EPstat’s usefulness and potential for improvement. The main limitation is the small sample of survey respondents. There was probably nonresponse bias in that nonresponders may be different from responders. While this is acceptable for a qualitative analysis, it clearly affects generalizability. The small sample also limits analysis of variations within the data. To partly counter this, and because the investigators mainly have a tertiary center perspective, we conducted the workshops and made sure to recruit participants representing multiple clinics in our region. As the project progresses, larger evaluations are needed with methodological improvement. Additionally, the survey instrument was not formally validated; however, we believe that the questions were intuitive and easy for our colleagues to understand. We would also like to conduct larger and even more representative qualitative workshops with improved sampling of participants and content coded and thematically analyzed in a systematic manner. Such workshops are under preparation together with an external partner evaluating health care digitalization.

EPstat is a digital and data-driven decision support tool and, thus, is similar to future possible AI tools (trained on patient data but then generating their own suggested clinical decisions). Although it is technically distinct from such solutions, EPstat can clearly provide insights on user adaptation and how such tools will be received by health services. Therefore, experiences from the introduction of EPstat, we hope, are likely to provide information that is valuable for the introduction of AI tools. Specifically in epilepsy, it may also be useful as a platform for distribution of such tools.

In conclusion, this study highlights EPstat’s potential to improve epilepsy care in general and ASM selection in particular. This is also the first example of big data being looped back to the clinic to improve decision-making, making it conceptually interesting. Survey respondents generally perceived the platform as user-friendly and relevant, but only a few had tried it—which is an important lesson for the future development of tools, including AI, that provide decision support. To make the platform more appealing and attract more users, the current offering could be expanded; easily accomplished additions would be to provide summaries of important drug information and checklists for epilepsy management. Long-term sustainability would require an automatic (or semiautomatic) feedback process with continuous updates to maintain the platform’s relevance. Other potential long-term developments include broadening the scope of the data to encompass the third ASM and incorporating data from neighboring countries with comparable data sources. Important next steps will be a sustainable ownership and integration into the electronic health record system in the region. Mobile access is another important step as sometimes ASMs are recommended by neurologists being called for advice by physicians in other specialties (not neurology outpatient clinics). Cross-national data linkage would be a significant advantage if it could be achieved—vastly increasing the precision of the calculated retention rates and providing more rapid information on early use of new ASMs; if a drug is very well retained, that would become evident faster if many countries contributed with data. In 2025, we started a regional awareness campaign and investigation of how the ASM prescription pattern changed since EPstat’s initial launch. If this provides proof of impact, development will continue.

## Abbreviations

ASM: antiseizure medication
